# Dopamine Enhances Model-Based over Model-Free Choice Behavior

**DOI:** 10.1016/j.neuron.2012.03.042

**Published:** 2012-08-09

**Authors:** Klaus Wunderlich, Peter Smittenaar, Raymond J. Dolan

**Affiliations:** 1 Wellcome Trust Centre for Neuroimaging, University College London, London WC1N 3BG, UK

## Abstract

Decision making is often considered to arise out of contributions from a model-free habitual system and a model-based goal-directed system. Here, we investigated the effect of a dopamine manipulation on the degree to which either system contributes to instrumental behavior in a two-stage Markov decision task, which has been shown to discriminate model-free from model-based control. We found increased dopamine levels promote model-based over model-free choice.

## Highlights

► Dopamine increases relative degree of model-based to model-free behavior

## Teaser

Decision making arises out of contributions from model-free habitual and model-based goal-directed systems. Wunderlich et al. investigate how dopamine affects each system to contribute to instrumental behavior, finding that increased dopamine levels preferentially promote model-based goal-directed choice.

## Introduction

An overarching view of adaptive behavior is that humans and animals act to maximize reward and minimize punishment as a consequence of their choices. There are multiple ways this can be realized, and mounting evidence indicates model-based and model-free forms of reinforcement learning (RL) contribute to behavioral control (Balleine and O'Doherty, 2010; Boureau and Dayan, 2011; Daw et al., 2005; Doya, 1999; Redgrave et al., 2010; Wunderlich et al., 2012). Model-free RL learns the course of action leading to maximum long-run reward through a temporal difference (TD) prediction error teaching signal (Montague et al., 1996). By comparison, model-based choice involves forward planning, in which an agent searches a cognitive model of the environment to find the same optimal actions (Dickinson and Balleine, 2002).

An unresolved question is whether neuromodulatory systems implicated in value-based decision making, specifically dopamine, impact on the degree to which one or the other controller is dominant in choice behavior. Phasic firing of dopaminergic VTA neurons encodes reward prediction errors in reinforcement learning (Hollerman and Schultz, 1998; Schultz et al., 1997). In humans, drugs enhancing dopaminergic function (e.g., L-DOPA) augment a striatal signal that expresses reward prediction errors during instrumental learning and, in so doing, increases the likelihood of choosing stimuli associated with greater monetary gains (Bódi et al., 2009; Frank et al., 2004; Pessiglione et al., 2006).

While previous research has focused on the role of dopamine in model-free learning, and value updating via reward prediction errors, its role in model-based choice remains poorly understood. For example, it is unknown if and how dopamine impacts on performance in model-based decisions and on the arbitration between model-based and model-free controllers. This is the question we address in the present study, in which we formally test whether dopamine influences the degree to which behavior is governed by either control system.

## Results

We studied 18 subjects on a two-stage Markov decision task after being treated with Madopar (150 mg L-DOPA plus 37.5 mg benserazide) or a placebo in a double-blind, fully counterbalanced, repeated-measures design. We used a task previously shown to distinguish model-based and model-free components of human behavior and in which subjects' choices pertain to a mixture of both systems (Daw et al., 2011). These properties render this task optimally suited to test the influence of a pharmacological manipulation on the degree to which choice performance expresses model-based or model-free control.

**Figure 1:**
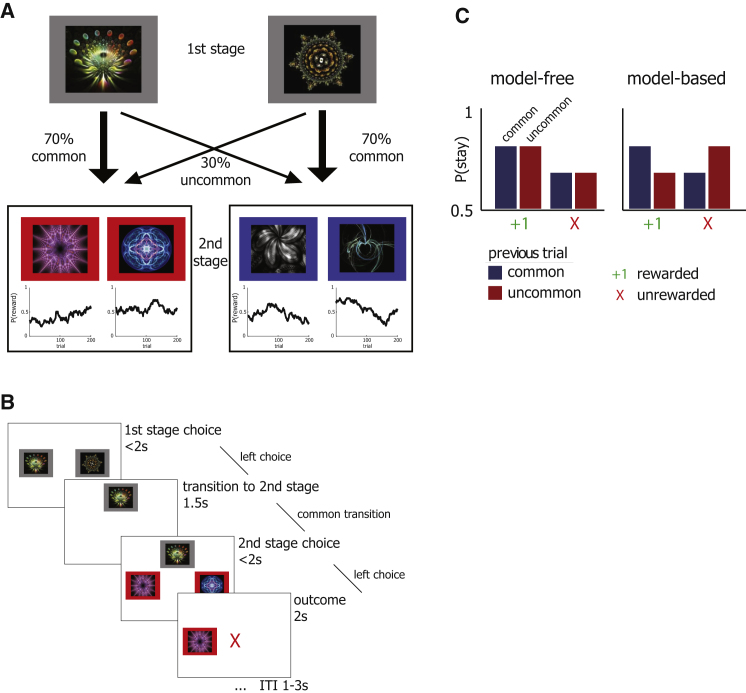
Task Design Task. (A) On every trial, a choice between two stimuli (left-right randomized) led probabilistically to one of two second-stage states, each of which then demanded another choice between two different stimulus pairs. Importantly, each first-stage stimulus was more strongly (70% versus 30%) associated with a particular second-stage state throughout the experiment, imposing a task structure that could be exploited in model-based choice. All stimuli in stage 2 were associated with probabilistic reward, which changed slowly and independently according to Gaussian random walks. This forced subjects to continuously learn and explore the second-stage choices throughout the experiment. (B) Timings in a single trial. (C) Model-based and model-free strategies for RL predict different patterns by which outcomes experienced after the second stage should impact first-stage choices on subsequent trials (based on Daw et al., 2011). If choices were driven by the model-free system, then a reward should increase the likelihood of choosing the same stimulus on the next trial, regardless of the type of transition (left). Alternatively, if choices were driven by a model-based system, we would expect an interaction between transition type and reward (right).

In each trial, subjects made an initial choice between two fractal stimuli, leading to either of two second-stage states in which they made another choice between two different stimuli (see Figures 1A and 1B). Each of the four second-stage stimuli was associated with probabilistic monetary reward. To incentivize subjects to continue learning throughout the task, we changed these probabilities slowly and independently according to Gaussian random walks. The choice of each stimulus on the first stage led predominantly (70% of the time) to one of the two associated second-stage states, a relationship that was fixed throughout the experiment. The logic of the task was that a dependence on model-based or model-free strategies predicts different patterns by which feedback obtained after the second stage should impact future first-stage choices.

We first considered stay-switch behavior as a minimally constrained approach to dissociate model-based and model-free control. A model-free reinforcement learning strategy predicts a main effect of reward on stay probability. This is because model-free choice works without considering structure in the environment; hence, rewarded choices are more likely to be repeated, regardless of whether that reward followed a common or rare transition. A reward after an uncommon transition would therefore adversely increase the value of the chosen first-stage cue without updating the value of the unchosen cue. In contrast, under a model-based strategy, we expect a crossover interaction between the two factors, because a rare transition inverts the effect of a subsequent reward (Figure 1C). Under model-based control, receiving a reward after an uncommon transition increases the propensity to switch. This is because the rewarded second-stage stimulus can be more reliably accessed by choosing the rejected first-stage cue than by choosing the same cue again.

**Figure 2:**
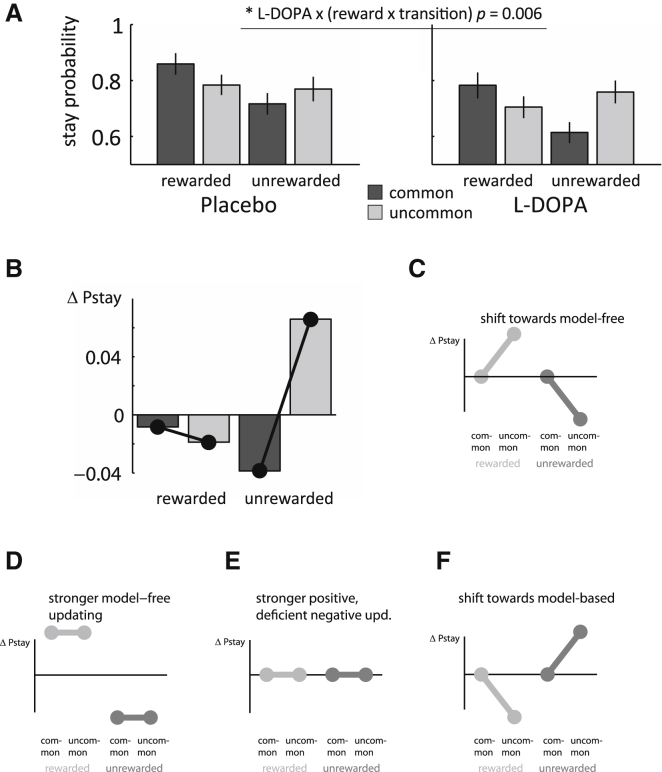
Results Stay-Switch Analysis (A) Subjects' task behavior showed characteristics of both model-free and model-based influences, demonstrating that subjects combined both strategies in the task. The reward × transition interaction (a measure of the extent to which subjects consider the task structure) was significantly larger in L-DOPA compared to placebo, indicating stronger model-based behavior. Error bars represent SEM. (B) Difference in stay probability between L-DOPA and placebo condition, corrected for the main effect of drug. The observed interaction indicates a shift toward model-based choice (see F) but shows no resemblance to any of the three effects implicating the model-free system (see C–E). (C–F) Illustration of expected differences in stay probability for hypothetical drug effects. See Figure S1 and Table S1 for validation of these hypotheses. (C) Trials after uncommon transitions (second and fourth bar) are discriminatory between model-free and model-based choice, whereas both models make equal predictions for trials after common transitions (cf. Figure 1C). A shift toward model-free control would be indicated by an increased propensity to stay with the chosen pattern after uncommon rewarded trials and an increase in switching after uncommon unrewarded trials. (D) Stronger or faster model-free learning would increase the reward-dependent effect and be expressed as a general increase to stay after rewarded trials and general decrease to stay after unrewarded trials. (E) A selective enhancement of positive updating paired with impairment in negative updating might not change mean-corrected stay probabilities. This is because enhanced positive updating leads to a stronger propensity to stay after rewarded trials, while impaired updating of unrewarded trials decreases the propensity to switch after such trials. (F) Opposite to (C), a shift toward model-based control is expressed by enhanced sensitivity to the task structure.

Using repeated-measures ANOVA, we examined the probability of staying or switching at the first stage dependent on drug state (L-DOPA or placebo), reward on previous trial (reward or no reward), and transition type on previous trial (common or uncommon) (see Figure 2A). A significant main effect of reward, *F*(1,17) = 23.3, p < 0.001, demonstrates a model-free component in behavior (i.e., reward increases stay probability regardless of the transition type). A significant interaction between reward and transition, *F*(1,17) = 9.75, p = 0.006, reveals a model-based component (i.e., subjects also take the task structure into account). These results show both a direct reinforcement effect (model-free) and an effect of task structure (model-based) and replicate previous findings (Daw et al., 2011).

The key analyses here concerned whether L-DOPA modulated choice propensities. Critically, we observed a significant drug × reward × transition interaction, *F*(1,17) = 9.86, p = 0.006, reflecting increased model-based behavior under L-DOPA treatment. We also observed a main effect of the drug, *F*(1,17) = 7.04, p = 0.017, showing that subjects are less perseverative under L-DOPA treatment. Interactions between drug and transition, *F*(1,17) = 4.09, p = 0.06, or drug and reward (which would indicate a drug-induced change in model-free control), *F*(1,17) = 1.10, p = 0.31, were not significant.

Figure 2B shows the difference in stay probability between drug states corrected for a main effect of drug. Note that dopamine treatment particularly affected choices after unrewarded trials and a post hoc contrast; testing for a differential drug effect after unrewarded compared to rewarded trials confirmed this was significant, *F*(1,17) = 12.68, p = 0.002. Figures 2C–2F illustrate how a number of hypothesized effects of L-DOPA might manifest itself in a stay-switch analysis (see Figure S1 available online for a validation of these hypotheses using computational modeling). Qualitatively, the data in Figure 2B resemble a shift toward model-based control, most notable after unrewarded trials. In contrast, our results do not resemble any of the putative model hypotheses that invoke modulation of a model-free system.

Given the broad effects of drug in this analysis, we next employed computational modeling to provide an in-depth understanding of this pharmacological effect. The value of using such an approach is that a stay-switch analysis only considers variables on trial n − 1, while a computational model encompasses an integration over a longer reward history and attributes any behavioral change to a specific computational process.

Model comparisons (Table S2) between a fully parameterized hybrid model (Daw et al., 2011; Gläscher et al., 2010) and various reduced nested versions favored a model with the parameters learning rate α, softmax temperature β, perseverance π, and relative degree of model-based/model-free control ω as best fit. We then fitted parameters individually for each subject and drug state after applying logistic or exponential transformations to bounded model parameters (*α*, *β*, *π*, *ω*) to gain Gaussian distributed fitted parameter values (*a*, *b*, *p*, *w*), permitting the use of parametric tests for differences between sessions. All reported p values are from two-tailed paired t tests.

**Figure 3:**
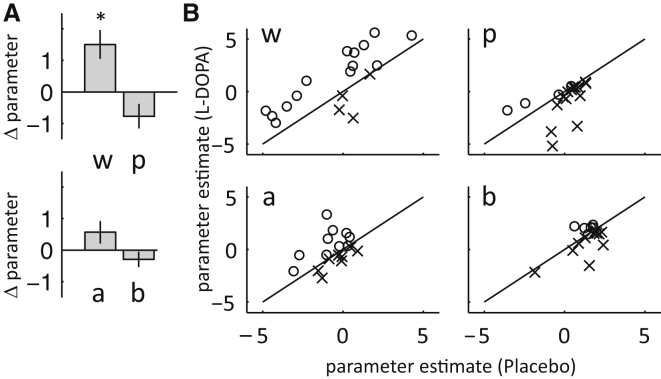
Results Computational Learning Model (A) Group-averaged parameter differences in the computational learning model between L-DOPA and placebo. Parameter *w* represents a measure of model-based over model-free control. Error bars represent SEM. (B) Single-subject data for parameter values in (A). Each data point represents the parameter value of a single subject. Subjects above the diagonal (circles) had higher parameter values in the L-DOPA compared to placebo condition, while subjects below (crosses) had smaller parameter values. The relative degree of model-based control was higher in the L-DOPA condition in 14 out of 18 subjects. See Figure S1 and Table S1 for validation of the winning model and Table S2 for model comparison details.

In line with the stay-switch results, we found a significant increase in the model-based weighting parameter *w*, p = 0.005, (positive in 14 out of 18 subjects) and a trend-level decrease in the perseverance parameter *p*, p = 0.06, under L-DOPA compared to placebo. Learning rate *a*, p = 0.45, and softmax temperature *b*, p = 0.34, did not differ between drug states (Figure 3). We note that, overall, fitted parameter values were in a similar range as those in Daw et al. (2011) (Table 1). As model-based choice is superior to model-free choice in this task, we found a significant positive correlation between subjects' relative degree of model-based control (*w*) and total earnings, r = 0.4, p = 0.01 (Figure S2). There was no evidence for differences in drowsiness or general alertness (Bond et al., 1974) between sessions (paired t tests over each score; smallest p > 0.1) or in average response times between drug states (first stage RT_L-DOPA_ = 593 ms, RT_Placebo_ = 586 ms; paired t test, p = 0.70).

Note that in the preceding analysis we employed the same computational models as the authors in the original study utilizing this task (Daw et al., 2011). We also constructed additional computational models to further explore the observed shift in control and to examine whether dopamine asserts its effect predominantly on the model-free or model-based system. Some studies have suggested that dopamine levels might have differential effects on positive and negative updating (Frank et al., 2004; Pessiglione et al., 2006). We therefore tested a model with separate learning rates for positive (a+) and negative (a−) updating. The learning rates were not significantly different between L-DOPA and placebo (paired t test: a+, p = 0.52 and a−, p = 0.43). The use of the same values at the second stage for both model-free and model-based systems ignores evidence that model-based and model-free learning use different neural structures (Balleine and O'Doherty, 2010; Wunderlich et al., 2012) and, as such, might learn the second-stage values separately. To test this, we implemented a model containing separate representations of second-stage values and learning rates for the model-based and model-free system. The model-based learning rate was higher than the model-free learning rate (p = 0.001). However, concurring with the results from our original computational implementation, there was no change in either learning rate with drug condition (α model-free p = 0.33, model-based p = 0.76). An alternative computational implementation of model-free RL, the actor-critic model, learns values and action policies separately (Sutton and Barto, 1998). To test whether L-DOPA might alter updating of action policies rather than impacting on value updating, we implemented a hybrid model in which the original model-free component was replaced with an actor-critic component. In line with the absence of a significant difference in the parameters of the original model-free implementation, this analysis did not show any significant difference between drug states in either the learning parameter α (p = 0.17) for state value or η for policy updating (p = 0.51).

Finally, we tested for order effects by repeating the analyses with session instead of drug as factor. There were no significant differences in either stay-switch behavior (repeated-measures ANOVA; main effect of session F(1,17) < 1; session × reward, *F*(1,17) < 1; session × (reward × transition), *F*(1,17) = 1.37, p = 0.26) or parameter fits in the computational analysis with session as a grouping factor (two-tailed paired t tests; *a*: p = 0.15; *b*: p = 0.31; *p*: p = 0.97; *w*: p = 0.37). Thus, our results provide compelling evidence for an increase in the relative degree of model-based behavioral control under conditions of elevated dopamine.

## Discussion

It is widely believed that both model-free and model-based mechanisms contribute to human choice behavior. In this study, we investigated a modulatory role of dopamine in the arbitration between these two systems and provide evidence that L-DOPA increases the relative degree of model-based over model-free behavioral control.

The use of systemic L-DOPA combined with a purely behavioral approach precludes strong conclusions about the precise physiological underpinnings of the observed shift to model-based control. Nevertheless, we provide a number of possible explanations for how this effect might be mediated in the brain that could guide further studies. First, increased dopamine levels may improve performance of component processes of a model-based system. Dopamine has previously been associated with an enhancement of cognitive functions such as reasoning, rule learning, set shifting, planning, and working memory (Clatworthy et al., 2009; Cools and D'Esposito, 2011; Cools et al., 2002; Lewis et al., 2005; Mehta et al., 2005), and these processes are most likely coopted during model-based decisions. Previous theoretical considerations link a system's performance to its relative impact on behavioral control, such that the degree of model-based versus model-free control depends directly on the relative certainties of both systems (Daw et al., 2005). Increased processing capacity might enhance certainty in the model-based system and would thus predict the observed shift in behavioral control that we detail here.

Second, a more conventional account is that increased dopamine exerts its effect through an impact on a model-free system. According to this view, excessive dopamine disrupts model-free reinforcement learning, which is then compensated for by increased model-based control. Specifically, elevated tonic dopamine levels may reduce the effectiveness of negative prediction errors (Frank et al., 2004; Voon et al., 2010). However, this explanation fails to account for the results presented here. First, a disruption of negative prediction errors under L-DOPA would change stay probabilities independent of transition type (Figure 2E), which is incompatible with the drug × reward × transition interaction observed here (Figure 2B). Second, any such model-free impairment would have impacted learning of second-stage values (which in this task are assumed to be learnt via prediction errors irrespective of the control on the first stage; Daw et al., 2011) and manifested in noisier choices or altered learning rates. We did not observe such an effect on the softmax temperature *b* or learning rate *a*. This effect was still absent when we fit alternative models employing separate learning rates and temperatures for the first and second stage or separate learning rates for positive and negative updating. Together, this argues against the idea that L-DOPA in our study enhanced the relative degree of model-based behavior through a disruption of the model-free system.

Finally, dopamine could facilitate switching from one type of control to the other akin to the way it decreases behavioral persistence (Cools et al., 2003). It is known that over the course of instrumental learning, the habitual system assumes control from the goal-directed system (Adams, 1982; Yin et al., 2004), but the goal-directed system can quickly regain control in unforeseen situations (Isoda and Hikosaka, 2011; Norman and Shallice, 1986). This could explain why we observe a stronger switch to model-based behavior after unrewarded trials: the lack of rewarding feedback may prompt the need to reevaluate available options and invest more energy to prevent another nonrewarding event by switching to model-based control. Note that it is possible and indeed likely that a facilitation of control switching under L-DOPA works in concert with an enhancement of the model-based system itself.

The predominant view in computational and systems neuroscience holds that phasic dopamine underlies model-free behavior by encoding reward prediction errors. On the other hand, animal and cognitive approaches emphasize a role for dopamine in model-based behavior such as planning and reasoning (Berridge, 2007; Clatworthy et al., 2009; Cools and D'Esposito, 2011; Robbins and Everitt, 2007). Contrasting with interest in the model-free and model-based system separately is the lack of data on the arbitration between these two behavioral controllers. Our experiment fills this gap by pitting model-free and model-based control against each other in the same task and in so doing provides strong evidence for an involvement of dopamine in the arbitration between model-free and model-based control over behavior.

Our findings advocate an effect of L-DOPA on the arbitration between model-based and model-free control, without a modulation of the model-free system itself. Note that the majority of studies reporting enhanced or impaired learning under dopaminergic drugs used either Parkinson's disease (PD) patients (Frank et al., 2004; Voon et al., 2010) or involved agents that primarily act at D2 receptors (Cools, 2006; Frank and O'Reilly, 2006). In contrast with these studies, we did not find evidence for any modulation by L-DOPA of model-free learning rates or indeed evidence of impaired model-free choices. These deviations might partly be explained by PD patients' more severely reduced dopamine availability off their dopamine replacement therapy (in contrast to our placebo condition) and the much higher doses of medication involved in PD treatment. Consistent with this explanation is that the effect of L-DOPA on instrumental learning in healthy volunteers was found to be significant only when compared to an inhibition of the dopamine system (via haloperidol) but not when compared to placebo (Pessiglione et al., 2006).

Our task does not allow us to dissociate between learning and performance effects. Previous work has suggested interactions between model-based and model-free systems during learning. In this framework, reward prediction errors that are in line with model-based predictions are enhanced, while reward prediction errors that are in opposition with model-based predictions are attenuated (confirmation bias) (Biele et al., 2011; Doll et al., 2009, 2011). In support of this, neuroimaging findings based on the present task showed evidence that ventral striatal BOLD at the time of feedback, typically associated with prediction error signals, contained a model-based component (Daw et al., 2011). This raises the possibility that model-free and model-based systems are not segregated systems whose influence is weighted at the time of choice. Instead, choices could also be made by a model-free system in which learning is modulated by transition probabilities. In this study, we cannot unambiguously differentiate between these accounts and further fine-grained investigations, in part motivated by the present data, are required to understand this complex issue.

Dopamine itself is a precursor to norepinephrine and epinephrine, potentially contributing to the observed effects. However, L-DOPA administration causes a linear increase in dopamine levels in the brain without affecting norepinephrine levels (Everett and Borcherding, 1970). Another possibility would be that L-DOPA exerts effects through interactions with the serotonin system. Such an interaction, between dopamine and serotonin, is known to play a role in a range of higher-level cognitive functions (Boureau and Dayan, 2011).

By implicating dopamine in behavioral control, we open the door to further experiments aimed at elucidating the precise neural mechanisms underlying the arbitration between both controllers. While theoretical considerations afford a number of ways for how this arbitration might be implemented in the brain (Daw, 2011; Keramati et al., 2011), our results provide empirical evidence that dopamine influences the relative degree between model-free and model-based control.

## Experimental Procedures

### Subjects

Eighteen healthy males (mean age: 23.3 [SD: 3.4]) participated in two separate sessions. Data from two additional subjects were not included in the analysis as those subjects misunderstood instructions and performed at chance level. The UCL Ethics committee approved the study and subjects gave written informed consent before both sessions.

### Dopamine Drug Manipulation

Subjects were tested in a double-blind, fully counterbalanced, repeated-measures setting on L-DOPA (150 mg _L_-3,4-dihydroxyphenylalanine / 37.5 mg benserazide; Madopar, Roche) and on placebo (500 mg calcium carbonate; Calcit, Procter and Gamble) dispersed in orange squash. The task was administered 55.0 (SD: 4.7) min after drug administration. Sessions one and two were approximately 1 week apart (at least 4, but no more than 14 days), with both sessions at the same time of day. All subjects except one participated in the morning to minimize time-of-day effects. We assessed drug effects on self-reported mental state using a computerized visual analog scale immediately before starting the task (Bond et al., 1974).

### Task

We drew on Daw et al. (2011)'s two-step choice task to assess the relative degree of model-based versus model-free decision making. Our version of the task was identical to Daw et al.'s except for different stimulus images (semantically irrelevant fractals), a slightly larger dynamic range of reward probabilities, and more rapid trial timings. Subjects completed 201 trials and were given a break after trials 67 and 134. Please see Supplemental Experimental Procedures for full task description.

### Stay-Switch Behavior

Stay probabilities at the first stage (the probability to choose the same stimulus as in the preceding trial), conditional on transition type of the previous trial (common or uncommon), reward on the previous trial (reward or no reward), and drug state (L-DOPA or placebo) were entered into a three-way repeated-measures ANOVA.

### Computational Modeling

We fit a previously described hybrid model (Gläscher et al., 2010; Daw et al., 2011) to choice behavior. This model contains separate terms for model-free and model-based stimulus values at the first stage. These values are weighted by a parameter *w* to compute an overall value for each stimulus. The first-stage choice is then made using a softmax function dependent on relative stimulus values and the subject's choice on the previous trial. For a full description of the model, see Supplemental Experimental Procedures.

### Model Fitting

We used a hierarchical model-fitting strategy, which takes into account the likelihood of individual subject choices *c*_*i*_ given the individual subject parameters *a*_*i*_, *b*_*i*_, *p*_*i*_, *w*_*i*_ and also the likelihood of the individual subject parameters given the parameter distribution in the overall population across conditions. This regularizes individual subjects' parameter fits, rendering them more robust toward overfitting.

This two-stage hierarchical procedure is a simplified estimation strategy of the iterative expectation − maximization (EM) algorithm (see Supplemental Experimental Procedures for details, and for an in-depth discussion see also Daw, 2011).

Importantly, our main results are independent of the parameter regularization: the weighting parameter *w* was significantly (p = 0.02) higher in the L-DOPA condition compared to placebo, even when testing individual subject parameters from the maximum likelihood fit during the first step.

### Parameter Covariance

Covariance between parameters would indicate that two parameters might be redundant, potentially rendering parameter values more difficult to interpret. There were no significant pairwise correlations between any of our parameters across subjects (paired t tests: all individual p > 0.05).

## Figures and Tables

**Table 1:** Best-Fitting Parameter Estimates, Shown Separately for Both Drug Conditions as Median and Quartiles across Subjects

	α	β	π	ω
**Placebo**				

25th percentile	0.25	3.4	0.63	0.07
Median	0.45	5.4	1.37	0.58
75th percentile	0.57	7.3	2.20	0.79

**L-DOPA**				

25th percentile	0.25	1.8	0.28	0.14
Median	0.37	4.7	0.70	0.78
75th percentile	0.59	7.7	1.50	0.95

## Supplemental Information

Document S1. Figures S1 and S2, Tables S1 and S2, and Supplemental Experimental Procedures

## References

[bib1] Adams C.M. (1982). Variations in the sensitivity of instrumental responding to reinforcer devaluation. Q. J. Exp. Psychol..

[bib2] Balleine B.W., O'Doherty J.P. (2010). Human and rodent homologies in action control: corticostriatal determinants of goal-directed and habitual action. Neuropsychopharmacology.

[bib3] Berridge K.C. (2007). The debate over dopamine's role in reward: the case for incentive salience. Psychopharmacology (Berl.).

[bib4] Biele G., Rieskamp J., Krugel L.K., Heekeren H.R. (2011). The neural basis of following advice. PLoS Biol..

[bib5] Bódi N., Kéri S., Nagy H., Moustafa A., Myers C.E., Daw N., Dibó G., Takáts A., Bereczki D., Gluck M.A. (2009). Reward-learning and the novelty-seeking personality: a between- and within-subjects study of the effects of dopamine agonists on young Parkinson's patients. Brain.

[bib6] Bond A.J., James D.C., Lader M.H. (1974). Physiological and psychological measures in anxious patients. Psychol. Med..

[bib7] Boureau Y.L., Dayan P. (2011). Opponency revisited: competition and cooperation between dopamine and serotonin. Neuropsychopharmacology.

[bib8] Clatworthy P.L., Lewis S.J., Brichard L., Hong Y.T., Izquierdo D., Clark L., Cools R., Aigbirhio F.I., Baron J.C., Fryer T.D., Robbins T.W. (2009). Dopamine release in dissociable striatal subregions predicts the different effects of oral methylphenidate on reversal learning and spatial working memory. J. Neurosci..

[bib9] Cools R. (2006). Dopaminergic modulation of cognitive function-implications for L-DOPA treatment in Parkinson's disease. Neurosci. Biobehav. Rev..

[bib10] Cools R., D'Esposito M. (2011). Inverted-U-shaped dopamine actions on human working memory and cognitive control. Biol. Psychiatry.

[bib11] Cools R., Stefanova E., Barker R.A., Robbins T.W., Owen A.M. (2002). Dopaminergic modulation of high-level cognition in Parkinson's disease: the role of the prefrontal cortex revealed by PET. Brain.

[bib12] Cools R., Barker R.A., Sahakian B.J., Robbins T.W. (2003). L-Dopa medication remediates cognitive inflexibility, but increases impulsivity in patients with Parkinson's disease. Neuropsychologia.

[bib13] Daw N.D., Phelps E., Robbins T., Delgado M. (2011). Trial-by-trial data analysis using computational models. Affect, Learning and Decision Making. Attention and Performance.

[bib14] Daw N.D., Niv Y., Dayan P. (2005). Uncertainty-based competition between prefrontal and dorsolateral striatal systems for behavioral control. Nat. Neurosci..

[bib15] Daw N.D., Gershman S.J., Seymour B., Dayan P., Dolan R.J. (2011). Model-based influences on humans' choices and striatal prediction errors. Neuron.

[bib16] Dickinson A., Balleine B.W., Pashler H., Gallistel R. (2002). The role of learning in the operation of motivational systems. Stevens' Handbook of Experimental Psychology.

[bib17] Doll B.B., Jacobs W.J., Sanfey A.G., Frank M.J. (2009). Instructional control of reinforcement learning: a behavioral and neurocomputational investigation. Brain Res..

[bib18] Doll B.B., Hutchison K.E., Frank M.J. (2011). Dopaminergic genes predict individual differences in susceptibility to confirmation bias. J. Neurosci..

[bib19] Doya K. (1999). What are the computations of the cerebellum, the basal ganglia and the cerebral cortex?. Neural Netw..

[bib20] Everett G.M., Borcherding J.W. (1970). L-DOPA: effect on concentrations of dopamine, norepinephrine, and serotonin in brains of mice. Science.

[bib21] Frank M.J., O'Reilly R.C. (2006). A mechanistic account of striatal dopamine function in human cognition: psychopharmacological studies with cabergoline and haloperidol. Behav. Neurosci..

[bib22] Frank M.J., Seeberger L.C., O'Reilly R.C. (2004). By carrot or by stick: cognitive reinforcement learning in parkinsonism. Science.

[bib23] Gläscher J., Daw N., Dayan P., O'Doherty J.P. (2010). States versus rewards: dissociable neural prediction error signals underlying model-based and model-free reinforcement learning. Neuron.

[bib24] Hollerman J.R., Schultz W. (1998). Dopamine neurons report an error in the temporal prediction of reward during learning. Nat. Neurosci..

[bib25] Isoda M., Hikosaka O. (2011). Cortico-basal ganglia mechanisms for overcoming innate, habitual and motivational behaviors. Eur. J. Neurosci..

[bib26] Keramati M., Dezfouli A., Piray P. (2011). Speed/accuracy trade-off between the habitual and the goal-directed processes. PLoS Comput. Biol..

[bib27] Lewis S.J., Slabosz A., Robbins T.W., Barker R.A., Owen A.M. (2005). Dopaminergic basis for deficits in working memory but not attentional set-shifting in Parkinson's disease. Neuropsychologia.

[bib28] Mehta M.A., Gumaste D., Montgomery A.J., McTavish S.F., Grasby P.M. (2005). The effects of acute tyrosine and phenylalanine depletion on spatial working memory and planning in healthy volunteers are predicted by changes in striatal dopamine levels. Psychopharmacology (Berl.).

[bib29] Montague P.R., Dayan P., Sejnowski T.J. (1996). A framework for mesencephalic dopamine systems based on predictive Hebbian learning. J. Neurosci..

[bib30] Norman D., Shallice T., Davidson R., Schwartz G., Shapiro D. (1986). Attention to action: willed and automatic control of behavior. Consciousness and Self-Regulation. Advances in Research and Theory.

[bib31] Pessiglione M., Seymour B., Flandin G., Dolan R.J., Frith C.D. (2006). Dopamine-dependent prediction errors underpin reward-seeking behaviour in humans. Nature.

[bib32] Redgrave P., Rodriguez M., Smith Y., Rodriguez-Oroz M.C., Lehericy S., Bergman H., Agid Y., DeLong M.R., Obeso J.A. (2010). Goal-directed and habitual control in the basal ganglia: implications for Parkinson's disease. Nat. Rev. Neurosci..

[bib33] Robbins T.W., Everitt B.J. (2007). A role for mesencephalic dopamine in activation: commentary on Berridge (2006). Psychopharmacology (Berl.).

[bib34] Schultz W., Dayan P., Montague P.R. (1997). A neural substrate of prediction and reward. Science.

[bib35] Sutton R.S., Barto A.G. (1998). Reinforcement Learning: An Introduction.

[bib36] Voon V., Pessiglione M., Brezing C., Gallea C., Fernandez H.H., Dolan R.J., Hallett M. (2010). Mechanisms underlying dopamine-mediated reward bias in compulsive behaviors. Neuron.

[bib37] Wunderlich K., Dayan P., Dolan R.J. (2012). Mapping value based planning and extensively trained choice in the human brain. Nat. Neurosci..

[bib38] Yin H.H., Knowlton B.J., Balleine B.W. (2004). Lesions of dorsolateral striatum preserve outcome expectancy but disrupt habit formation in instrumental learning. Eur. J. Neurosci..

