# Supplementary material for: Dopamine Enhances Model-Based over Model-Free Choice Behavior
Source: Neuron. 2012 Aug 9;75(3-4):418–24. doi: 10.1016/j.neuron.2012.03.042 (PMC3417237; doi:10.1016/j.neuron.2012.03.042)
Supplement: Document S1. Figures S1 and S2, Tables S1 and S2, and Supplemental Experimental Procedures [file mmc1.pdf]

Neuron, Volume *75*

Supplemental Information

Dopamine Enhances Model-Based over Model-Free Choice Behavior

Klaus Wunderlich, Peter Smittenaar, and Raymond J. Dolan

Figure S1

To confirm that our winning model can capture our key behavioral findings (i.e. the drug*reward*transition interaction on stay-switch behavior) we generated data for 500 virtual subjects on this task using the best-fitting parameters. These data were then subjected to a stay-switch analysis. We found an identical pattern of effects in these generated data as observed empirically in our participants (Figure S1A). Most importantly, the data generated by the model showed a significant 3-way interaction, indicating that the model indeed captures key components of the data (see Table S1). Note that, as expected, the model did not replicate the asymmetry in rewarded versus unrewarded trials shown in Figure 2B.

The idealized hypotheses put forward for the stay-switch analysis in Figures 2C-2F were based on ideas derived from previous literature. To validate these hypotheses we generated choices for virtual subjects, but now with adjustments to parameters based on specific hypotheses (Figures S1B-S1D). Our key hypotheses are fully supported by these simulations, showing that the computational models capture the key behavioral signatures of model-free and model-based behavior.

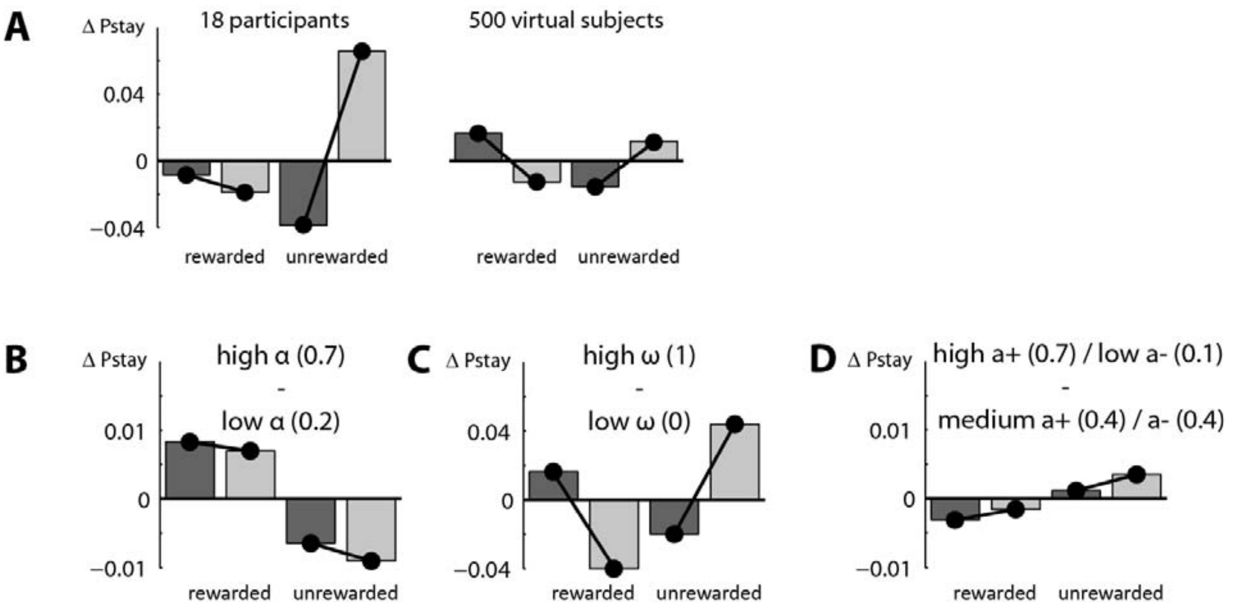

Figure S1. Validation of model and hypotheses (related to Figure 2). (**A**) Mean-corrected $P(stay)_{ON}$ - $P(stay)_{OFF}$ for 18 participants reported in the study (left) and 500 virtual subjects using best-fitting parameters in the winning model (right). (**B**) Modulation of the model-free learning rate α. A change in learning rate alters stay probability after rewarded versus unrewarded trials, but does not interact with transition. This is equivalent to Figure 2D in main text. (**C**) Model-based (ω = 1) versus model-free agent (ω = 0) shows a stronger reward*transition interaction. This is equivalent to Figure 2F in the main text. (**D**) Increase in positive learning rate and decrease in negative learning rate does not change relative stay probabilities, similar to our prediction in Figure 2E.

Figure S2

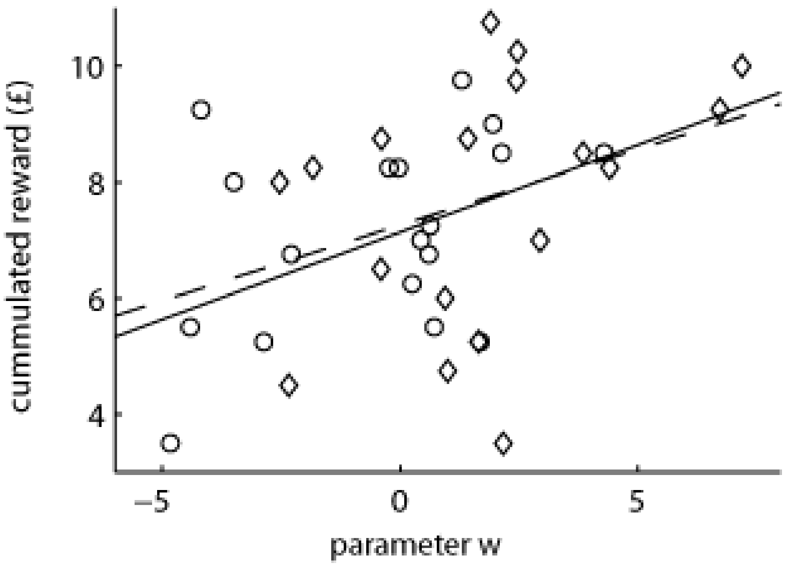

Figure S2. Task performance increases with degree of model-based control (related to Figure 3). Performance-based reward per session (£) correlated with degree of model-based control as indicated by the parameter fit *w* (r = 0.40, p = 0.01). This relation was still significant even when we control for the 3 other parameter values using partial correlations (r_{wr} = 0.34, p = 0.04). When we test for the correlation within each session, we find very similar regression coefficients for L-DOPA and placebo albeit each individual test is not significant due to the reduced statistical power (for placebo: r=0.4, p=0.10; for L-DOPA: r=0.39, p=0.12). Data points represent individual subjects and session (diamond: L-DOPA, circle: placebo). Regression lines plotted separately for L-DOPA (solid) and placebo (dashed).

Table S1. Statistical comparison of model-generated versus participant data (related to Figure 2)

Effect	18 participants		500 virtual subjects	
	F(1,17)	p	*F*(1,499)	p
drug	7.04	= .02	83.00	< .001
reward	23.30	< .001	6.01	= .02
transition	< 1	~	< 1	~
drug x reward	1.10	= .31	< 1	~
drug x transition	4.09	= .06	< 1	~
reward x transition	9.75	= .006	561.79	< .001
drug x reward x transition	9.86	= .006	16.62	< .001

The data generated by the model in Figure S1A was subjected to the same ANOVA as the participant data. The stay-switch data generated by the model showed the same effects as found in participants, most notably the three-way interaction that supports our claim that L-DOPA enhances model-based behavior. The model thus provides a reasonable account of the data. Identical patterns exist between the two datasets, given the statistical model used. Highlighting indicates significant effects.

Table S2. Model comparison (related to Table 1)

A. BIC scores

Model parameters	BIC	# parameters
α1, α2, β1, β2, λ, π, ω	7286	7
α1, α2, β1, β2, π, ω	7251	6
α, β1, β2, π, ω	7109	5
α1, α2, β, π, ω	7160	5
α, β, λ, π, ω	7164	5
α+, α−, β, π, ω	7192	**5**
**α, β, π, ω**	**7097**	**4**
α, β, ω	7846	3
α, β	8221	2
MF/MB learning rates	7018	5
Actor/critic learning	7308	5

B. Bayesian Model Comparison

	Placebo		L-DOPA	
Alternative model to α, β, π, ω	Better in #subjects	Exceedance probability	Better in #subjects	Exceedance probability
α1, α2, β1, β2, λ, π, ω	17	>0.999	15	0.999
α1, α2, β1, β2, π, ω	14	0.997	15	0.999
α, β1, β2, π, ω	13	0.970	14	0.998
α1, α2, β, π, ω	15	>0.999	15	>0.999
α+, α−, β, π, ω	12	>.831	15	>.996
α, β, λ, π, ω	16	>0.999	17	>0.999
α, β, ω	16	>0.999	12	0.944
α, β	16	>0.999	12	0.911
MF/MB learning rates	16	0.999	14	0.999
Actor/critic learning	18	>0.999	17	>0.999

A model with the parameters learning rate, softmax temperature, perseverance, and model-based weight fitted subjects' choices best in a model comparison that considers differences in model complexity. More complex model variants included separate model-free RL parameters for the first and second stage, and eligibility traces. A: the BIC scores are the Bayesian equivalent to a fixed effects analysis. B: the exceedance probability corresponds to a mixed effects analysis (Stephan et al., 2009).

We calculated posterior model probabilities for each subject and the group of subjects. In brief, the procedure by Stephan et al. rests on treating the model as a random variable and estimating the parameters of a Dirichlet distribution, which describes the probabilities for all models considered. These probabilities then define a multinomial distribution over model space, allowing one to compute how likely it is that a model generated the subjects' data. To decide which model is more likely, we use the conditional model probabilities to quantify an exceedance probability, i.e. a belief that a particular model is more likely than the other model, given the group data. Note that even though the average BIC of the model with separate representations of model-free/model-based (MF/MB) second stage values is slightly lower than the $\alpha, \beta, \pi, \omega$ model, random effects analysis shows that behavior in the majority of our subjects and data at the population level can be much better ($p>0.999$) explained by the $\alpha, \beta, \pi, \omega$ model. We therefore used this model for all analyses displayed in the figures.

Supplemental Experimental Procedures

Detailed task description

Each trial consisted of two stages, both requiring a choice between two stimuli. Each choice option was represented by a fractal in a colored box on a black background (Figure 1). At every choice, subjects had to respond within two seconds using the left/right cursor keys or the trial was aborted. Subjects rarely missed a trial [mean: 0.4%, SD: 1.5%], and those missed trials were omitted from analysis. Choice at the first stage always involved the same two stimuli left/right randomized. After subjects made their response the rejected stimulus disappeared from the screen and the chosen stimulus moved to the top of the screen. After 1.5 seconds one of two second stage stimulus pairs appeared, with the transition from first to second stage following fixed transition probabilities. Each first stage option was more strongly (with a 70% transition probability) associated with one of the two second stage pairs, a crucial factor in allowing us to distinguish model-free from model-based behavior (see below). After the second choice, the chosen option remained on the screen, together with a reward symbol (a pound coin) or a 'no reward' symbol (a red cross). Each of the 4 stimuli in stage 2 had a reward probability between 0.2 and 0.8. Those reward probabilities drifted slowly and independently for each of the four second stage options in every trial through a diffusion process with Gaussian noise (mean 0, SD 0.025).

Prior to the experiment, subjects were given explicit information about the task structure; namely that for each stimulus on the first stage one of the two transition probabilities was higher than the other, and that these transition probabilities remained constant throughout the experiment. Subjects were also told that reward probabilities on the second stage were independent of each other and would change slowly over time. To minimize the variance resulting from different outcome sequences we used the same two templates for outcome probabilities on all subjects. The assignment of templates to session and drug state was fully counterbalanced across subjects. On both days, subjects practiced 50 trials with different stimuli and outcome probabilities before starting the task. The main task consisted of 201 trials with short breaks after trial 67 and 134. Subjects' payout was related to a flat amount plus their overall accumulated rewards from both sessions (total range 16-30.40 in £s).

Computational modeling

In the following we denote the model-free value V_{s1}^{MF} and the model-based value V_{s1}^{MB} for first stage stimuli s1 $\in$ [1,2]. The hybrid model computes the actual value that is used in determining choice as weighted linear combination

$$V_{s1}^{Hybrid} = \omega * V_{s1}^{MB} + (1-\omega) * V_{s1}^{MF}. \quad (1)$$

Values for the four stimuli at the second stage (stimuli s2 $\in$ [3..6]) are updated identically for both models according to reward prediction errors (Rummery and Niranjan, 1994):

$$V_{s2}(t+1) = V_{s2}(t) + \alpha_2(r - V_{s2}(t)). \quad (2)$$

At the first stage, model-free 'cached' values are updated according to temporal difference learning with reward prediction errors and eligibility traces:

$V_{s1}^{MF}(t+1) = V_{s1}^{MF}(t) + \alpha_1(V_{s2\_chosen}(t) - V_{s1}^{MF}(t)) + \lambda\alpha_1(r - V_{s1}^{MF}(t))$, (3)

where α_1 / α_2 are learning rates at the first and second stage, and λ is a gain parameter for the eligibility traces.

Model-based values are calculated anew for each and every trial in a forward looking manner by multiplying the state values of the better option at the second stage with the state transition probabilities:

$V_1^{MB} = 0.7*\max(V_3, V_4) + 0.3*\max(V_5, V_6)$ and $V_2^{MB} = 0.3*\max(V_3, V_4) + 0.7*\max(V_5, V_6)$. (4)

Based on simulations by the authors of the original task we similarly simplified model-based learning by the premise that learning of state transitions quickly converges to stable values and hence we did not update transition probabilities by explicitly modeling state prediction errors (see supplementary materials in Daw et al. (2011) for a comprehensive discussion of this matter).

The probability *P* of choosing stimulus 1 (in a choice between stimulus 1 with value *V1* and stimulus 2 with value *V2*) was computed in stage 1 according to a softmax choice function dependent on the relative stimulus values and choice *C* in the previous trial.

$P(1) = 1 / (1 + \exp(-\beta_1(V1 - V2) -\pi(C1 - C2)))$ (5)

and similarly in stage 2

$P(1) = 1 / (1 + \exp(-\beta_2(V1 - V2)))$ (6)

For additional in-depth information on task and computational model see also Daw et al. (2011).

A model comparison using BIC scores (see below) of the full model with various reduced versions indicated that the best fitting model in the present experiment was a reduced model with single learning and softmax parameters for both stages, without the eligibility term (Table S2). This model includes variables for learning rates $\alpha_{1/2}$, inverse softmax temperatures $\beta_{1/2}$, perseverance π, and a parameter ω for the relative degree of model-based versus model-free control. Each of these parameters represents different aspects of choice behavior. The learning rate α captures the extent to which new information at outcome is used for learning, i.e. the learning speed; β measures the discriminability between two options, with a larger value pertaining to more precise choices when the values of alternative options are relatively close together; the persistency π is an index of the tendency to choose the same option as in the previous trial regardless of value (Kable and Glimcher, 2007), and parameter ω represents the extent to which one or other system drives a participant's behavior. By comparing ω across drug states we were able to examine how L-DOPA changes the relative importance of the model-free and model-based system in driving behavior in this task.

We applied logistic/exponential transformations before fitting parameters to transform bounded parameters into Gaussian distributed parameter values $x_i \sim N(\mu_x, \sigma_x)$, with population mean μ_x and

standard deviation σ_x. This transformation is justified by the premise that each individual subject (with parameter value x_i) is randomly drawn from a population of subjects with normally distributed parameters (with population mean μ_x and standard deviation σ_x) (Daw, 2011). It is important for our analysis because normally distributed parameter values permit the use of parametric tests and random effects statistics.

We transformed [0,1]-bounded α and ω into a Gaussian scale using the logistic function

$$\alpha = 1/(1+\exp(-a)), \text{ and } \omega = 1/(1+\exp(-w)) \quad (7)$$

and the logarithmically scaled β and π using the exponential function

$$\beta = \exp(b), \text{ and } \pi = \exp(p). \quad (8)$$

We denote model parameters by Greek letters and the Gaussian transformation by their respective Latin letters.

Hierarchical model fitting

A subject *i* drawn from the population has a set of parameters (i.e. a_i, b_i, p_i, w_i) according to a statistical distribution that characterizes the distribution of parameters in the population (with means μ_a, μ_b, μ_p, μ_w and standard deviations σ_a, σ_b, σ_p, σ_w). Adopting a model of the parameters in the population gives us a two-level hierarchical model of how a full dataset is produced. Each subject's parameters are drawn from population distributions, then the choice values and the observable choice data are generated according to the RL model with those parameters.

In a first pass we fitted parameters to every individual subject by maximizing the likelihood of subjects' choices given the parameterized model:

$$\begin{aligned} L &= P\left(c_i \mid \mu_a, \mu_b, \mu_p, \mu_w, \sigma_a, \sigma_b, \sigma_p, \sigma_w\right) = \\ &\int da_i db_i dp_i dw_i P(c_i \mid a_i, b_i, p_i, w_i) P(a_i \mid \mu_a, \sigma_a) P(b_i \mid \mu_b, \sigma_b) P(p_i \mid \mu_p, \sigma_p) P(w_i \mid \mu_w, \sigma_w) \end{aligned} \quad (9)$$

We next estimated mean and variance of the parameter distribution in the population based on our subject sample (e.g. $\mu_a = 1/N \sum_i^N a_i$ and $\sigma_a = \sqrt{\frac{1}{N} \sum_i^N (a_i - \mu_a)^2}$).

In a third step we refitted single subject parameter values by maximizing over both the likelihood of subjects' choices given the parameters and the likelihood for individual subject parameter values given the distribution of parameters in the population:

$$\begin{aligned} &P\left(a_i, b_i, p_i, w_i \mid c_i, \mu_a, \mu_b, \mu_p, \mu_w, \sigma_a, \sigma_b, \sigma_p, \sigma_w\right) \\ &\propto P\left(c_i \mid a_i b_i p_i w_i\right) \times P\left(a_i b_i p_i w_i \mid \mu_a, \mu_b, \mu_p, \mu_w, \sigma_a, \sigma_b, \sigma_p, \sigma_w\right) \end{aligned} \quad (10)$$

Model comparison

We performed model-selection between a fully parameterized hybrid model (accounting for learning rates and softmax choice temperatures separately at the first and second stage, and allowing for eligibility trace updating, perseverance, and a model-free versus model-based weighting parameter) and various reduced versions of this model by fitting the free parameters of each model across both sessions. Comparing Bayesian Information Criterion (BIC) (Schwarz, 1978), which considers differences in model complexity, we found a best fit for the model with a common learning rate and temperature for both stages, and a perseverance and weight parameter. We calculated BIC as

$$\mathrm{BIC} = 2L + k\ln(n) \qquad (11)$$

where L is the negative log likelihood function, n the number of choices and k the number of free model parameters.

Supplemental References

Balleine, B.W., and O'Doherty, J.P. (2010). Human and rodent homologies in action control: corticostriatal determinants of goal-directed and habitual action. Neuropsychopharmacology *35*, 48-69.

Biele, G., Rieskamp, J., Krugel, L.K., and Heekeren, H.R. (2011). The neural basis of following advice. PLoS Biol *9*, e1001089.

Daw, N.D. (2011). Trial-by-trial data analysis using computational models. In Affect, Learning and Decision Making. Attention and Performance., E. Phelps, T. Robbins, and M. Delgado, eds. (Oxford: Oxford University Press).

Daw, N.D., Gershman, S.J., Dayan, P., Seymour, B., and Dolan, R.J. (2011). Model-Based Influences on Humans' Choices and Striatal Prediction Errors. Neuron *69*, 1204-1215.

Doll, B.B., Hutchison, K.E., and Frank, M.J. (2011). Dopaminergic genes predict individual differences in susceptibility to confirmation bias. The Journal of neuroscience : the official journal of the Society for Neuroscience *31*, 6188-6198.

Frank, M.J., Seeberger, L.C., and O'Reilly R, C. (2004). By carrot or by stick: cognitive reinforcement learning in parkinsonism. Science *306*, 1940-1943.

Kable, J.W., and Glimcher, P.W. (2007). The neural correlates of subjective value during intertemporal choice. Nat Neurosci *10*, 1625-1633.

Pessiglione, M., Seymour, B., Flandin, G., Dolan, R.J., and Frith, C.D. (2006). Dopamine-dependent prediction errors underpin reward-seeking behaviour in humans. Nature *442*, 1042-1045.

Rummery, G.A., and Niranjan, M. (1994). On-Line Q-Learning Using Connectionist Systems. In CUEF/F-INFENG/TR.

Schwarz, G.E. (1978). Estimating the dimension of a model. Annals of Statistics *6*, 461-464.

Stephan, K.E., Penny, W.D., Daunizeau, J., Moran, R.J., and Friston, K.J. (2009). Bayesian model selection for group studies. Neuroimage *46*, 1004-1017.

Sutton, R.S., and Barto, A.G. (1998). Reinforcement Learning: An Introduction (MIT Press, Cambridge, MA).

Wunderlich, K., Dayan, P., and Dolan, R.J. (2012). Mapping value based planning and extensively trained choice in the human brain. Nature neuroscience *15*, 786–791.
